# Phylogenies from unaligned proteomes using sequence environments of amino acid residues

**DOI:** 10.1038/s41598-022-11370-x

**Published:** 2022-05-06

**Authors:** Juan Carlos Aledo

**Affiliations:** Department of Molecular Biology and Biochemistry, University of Málaga, 29071 Málaga, Spain

**Keywords:** Computational biology and bioinformatics, Evolution

## Abstract

Alignment-free methods for sequence comparison and phylogeny inference have attracted a great deal of attention in recent years. Several algorithms have been implemented in diverse software packages. Despite the great number of existing methods, most of them are based on word statistics. Although they propose different filtering and weighting strategies and explore different metrics, their performance may be limited by the phylogenetic signal preserved in these words. Herein, we present a different approach based on the species-specific amino acid neighborhood preferences. These differential preferences can be assessed in the context of vector spaces. In this way, a distance-based method to build phylogenies has been developed and implemented into an easy-to-use R package. Tests run on real-world datasets show that this method can reconstruct phylogenetic relationships with high accuracy, and often outperforms other alignment-free approaches. Furthermore, we present evidence that the new method can perform reliably on datasets formed by non-orthologous protein sequences, that is, the method not only does not require the identification of orthologous proteins, but also does not require their presence in the analyzed dataset. These results suggest that the neighborhood preference of amino acids conveys a phylogenetic signal that may be of great utility in phylogenomics.

## Introduction

It is a well-established fact that different genes from the same set of organisms often lead to different phylogenetic trees^1^. That happens even with mitochondrial-encoded genes^2,3^, despite the fact that such genes are inherited together without recombination, and the risk of confusing orthologous with paralogous sequences is non-existent. If, in addition, phenomena such as horizontal gene transfer, recombination, unrecognized paralogy, and highly variable rates of evolution are in place, the task of reconstructing accurate phylogenetic topologies can be seriously compromised. Not surprisingly, species phylogenies derived from comparison of single genes are seldom consistent with each other. To overcome this problem, two strategies are currently used when resolving phylogenies based on multiple alignments. In the so-called supermatrix approach, individual aligned genes or proteins are concatenated into a supermatrix, which is then subjected to phylogenetic analyses using either maximum likelihood or Bayesian inference^4^. In the alternative supertree method, gene or protein data sets are analyzed separately. Afterwards, the trees derived from these independent analyses are used to infer a single joined phylogeny^5,6^. Each of these alternatives has its own strengths and weaknesses, which has led to extensive discussions regarding the best strategy to conduct phylogenetic analyses of sequence data from multiple genes or proteins^7–10^. Nevertheless, both approaches have in common that they are time consuming, and they often require manual intervention. On the other hand, among the diverse sources of error in molecular phylogenies, incorrect sequence alignments rank high^11,12^. Therefore, those methods based on sequence alignments are prone to artefacts when used in phylogenomics^13^. Indeed, a number of previous studies have shown that the alignment method can have a considerable impact on tree topology^14–18^. Although attempts have been made to deal with multiple sequence alignment uncertainty during phylogeny reconstruction^19^, a satisfying and computationally tractable way to deal with alignment uncertainty is still lacking. Alignment artefacts have become even a bigger problem in the era of phylogenomics, where thousands of genes are automatically analyzed without accounting for alignment uncertainty^14^.

With the advent of modern genome sequencing techniques, it is now possible to consider phylogeny inference based on total genome sequences. However, given that most genomes contain millions of nucleotides, the standard approach based on positional homology (where each column from a multiple sequence alignment is considered as a homologous character) represents a daunting challenge that becomes impractical. Consequently, alternative approaches to compare whole genomes have been proposed. Thus, gene arrangement^20^, gene content^21,22^, protein domain-abundance^23^ and presence/absence of protein folds^24^, are all strategies tha have been explored to compare whole genomes. More recently, a wide number of alignment independent methods to compare sequences have been developed, and their utility in phylogenomics has been evaluated^25^. Thus, the so-called alignment-free approach include methods based on words-counting^26,27^, some of which implement diverse strategies to discriminate signal from noise^28–33^. Other published methods are based on matching statistics (i.e., they compute the length of common substrings with or without allowing mismatches)^34,35^, information theory^36,37^, splits driven by common subsequences^38^, or even based on micro-alignments^39,40^.

In this study, we describe a new and fast method for generating molecular phylogenies using multiple proteomes or protein-coding genomes. This method, which does not require sequence alignment or the identification of orthologous proteins, is based on a rationale previously unexplored in the context of phylogeny: the preference of each amino acid to be surrounded by other amino acids^41–43^. These species-specific preferences seem to posse a phylogenetic signal enough to reconstruct accurate tree topologies, even when the proteins analyzed from each species are functionally unrelated to the proteins selected from the other species.

## Results

**Figure 1:**
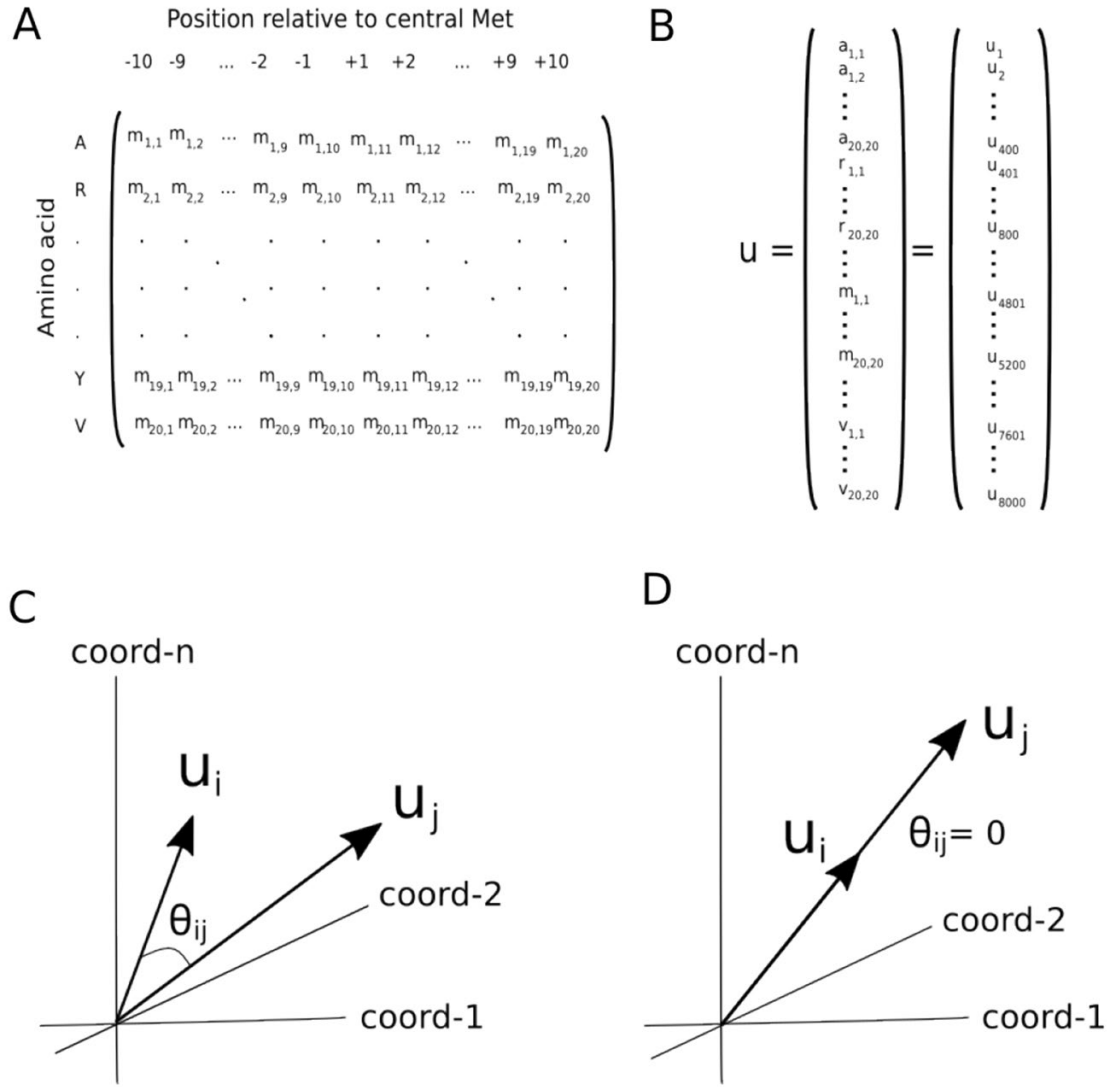
Encoding species as vectors. (**A**) For the sake of concreteness, we will focus on the sequence environment around methionine residues using a radius of 10 residues. Thus, a given proteome can be characterized by a matrix whose elements (*m*_*ij*_) provide the absolute frequency of the amino acid *i* at the position *j* in the environment of methionine residues. For instance, in this example, the element *m*_*2,2*_ gives the number of arginines found 9 residues away (toward the N-terminal) from any methionine residue. (**B**) Now, considering not just methionine but the 20 proteinogenic amino acids, the protein-coding genome of a given species of interest can be characterized by a set of 20 square matrices of order 20 or, equivalently, by a vector, $u\in {U}^{8000}$, of dimension 8000. It should be noted that, when coding species as vectors, the dimension of these vectors will depend on the radius chosen. Thus, in general, $u\in {U}^{n}$, where $n=800 radius$. (**C**) In this way, each vector is used to represent an organism (its protein-coding genome) within a set evolutionarily related species. (**D**) The direction, rather than the norm, of these vectors reflect the preference of the different amino acids at the different positions of the sequence environments. The work of encoding a set of genomes in a set of vectors is conveniently carried out by the function *otu.space()* from the R package accompanying the current paper.

**Figure 2:**
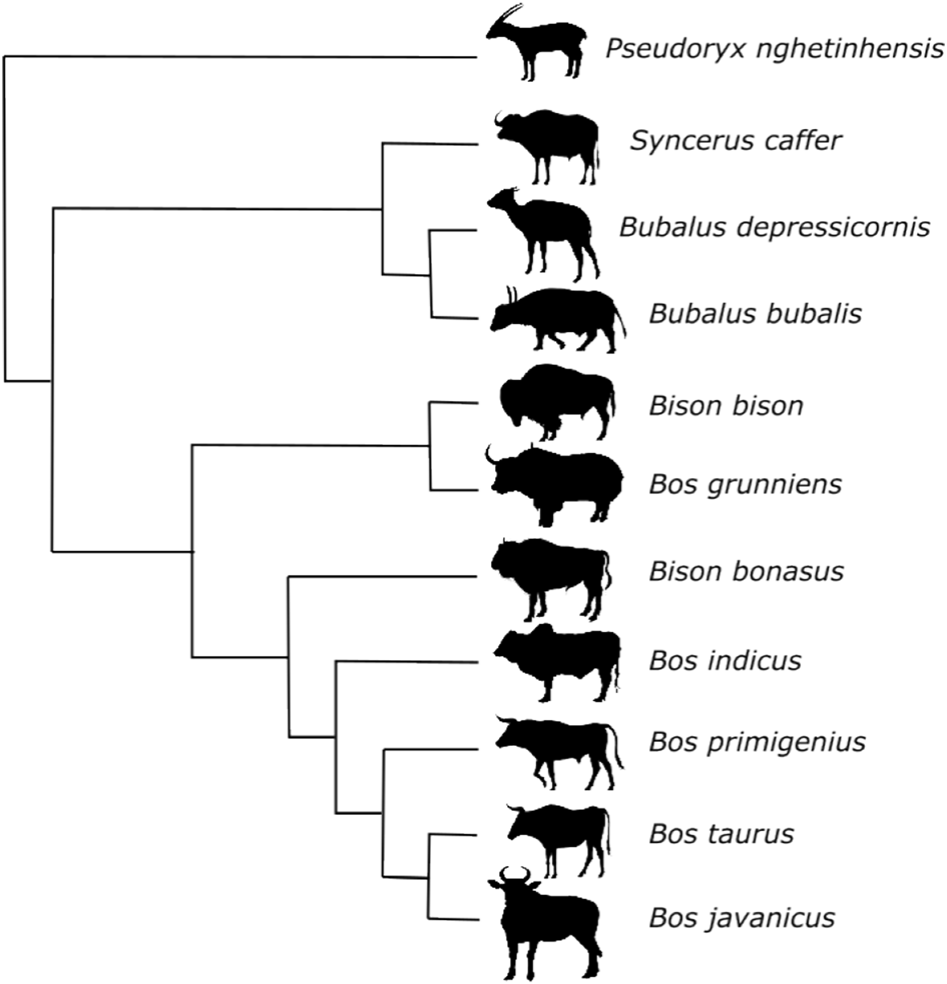
Molecular phylogeny of bovids. The phylogenetic relationships of 11 species of bovids were addressed using their protein-coded mitogenomes and different tree building methods (including classical alignment-based methods). Most methods produced the same tree topology shown in this figure.

The new method, which is presented in detail in the Methods section, is briefly outlined in Fig. 1. As a proof of concept, the phylogenetic relationships of 11 species of bovids were addressed using their protein-coding mitogenomes and the new method described below, hereinafter referred to as Env-NJ. The topology of the reconstructed tree is shown in Fig. 2. This topology fully matches that of the tree inferred using traditional alignment-based methods (reference tree), which reflect the accepted phylogeny for this groups of bovids. For comparative purposes, the same mitogenomes were employed with the following alignment-free tree-building packages: Feature Frequency Profile (FFP)^26^, alfpy (a stand-alone Phyton application that implements different approaches as well as different metrics to assess vectors distances)^27^, CVTree^32^, ALFRED-G^35^, SANS-serif^38^ and Prot-SpaM^39^. In all cases, the resulting trees were compared to the reference tree. Table 1 shows the corresponding normalized Robinson-Foulds distances (nRF).

**Table 1:** Normalized Robison-Foulds distances between trees obtained using different alignment-independent methods with respect to the refence tree shown in Fig. 2. The whole protein-coding mitogenomes of a group of 11 species of bovids were analyzed.

Approach	Package	Method	Language	nRF	Reference
Seq. environment	EnvNJ	cos, r = 10	R	0	Herein
Seq. environment	EnvNJ	jsd, r = 10	R	0	Herein
Seq. environment	EnvNJ	cheb, r = 10	R	0.38	Herein
k-mer counts	alfpy	cos, k = 5	Python	0.18	^25,27^
k-mer counts	alfpy	jsd, k = 5	Python	0	^25,27^
k-mer counts	alfpy	cheb, k = 5	Python	0.75	^25,27^
k-mer counts	FFP	jsd, k = 5	C	0	^26^
k-mer + f.p	^a^CVTree	cos, k = 5	C++	0	^32^
k-mer + f.p	^b^EnvNJ	SVD	R	0	^29^
k-mer + f.p	^c^alfpy	W-metric	Python	0.75	^25,27,33^
Matching statistics	ALFRED-G	cos, k = 5	C++	0.13	^35^
Splits/subseq	SANS-serif	strict, c = 2	C++	0.75	^38^
Information theory	alfpy	NCD	Python	0	^25,27^
Micro-alignments	Prot-SpaM	^d^(6, 40, 5)	C++	0.13	^39^

For details regarding the input parameters used for each method, please consult the given reference. Briefly, cos, jsd and cheb stand for cosine, Jensen-Shannon and Chebyshev metrics, respectively. The abbreviation f.p. stands for ‘further proccessing’. Thus (a) filters considering background words frequencies; (b) uses singular value decomposition, SVD, to analyze the 4-mer frequency data; (c) the matrix used for the W-metric analysis was Blosum62. (d) The parameters for the use of Prot-SpaM were weight of w = 6, with d = 40 (don’t-care positions) and m = 5 patterns. The parameter filter of SANS-serif was set to ‘strict’. NCD is the acronym of normalized compression distance.

**Figure 3:**
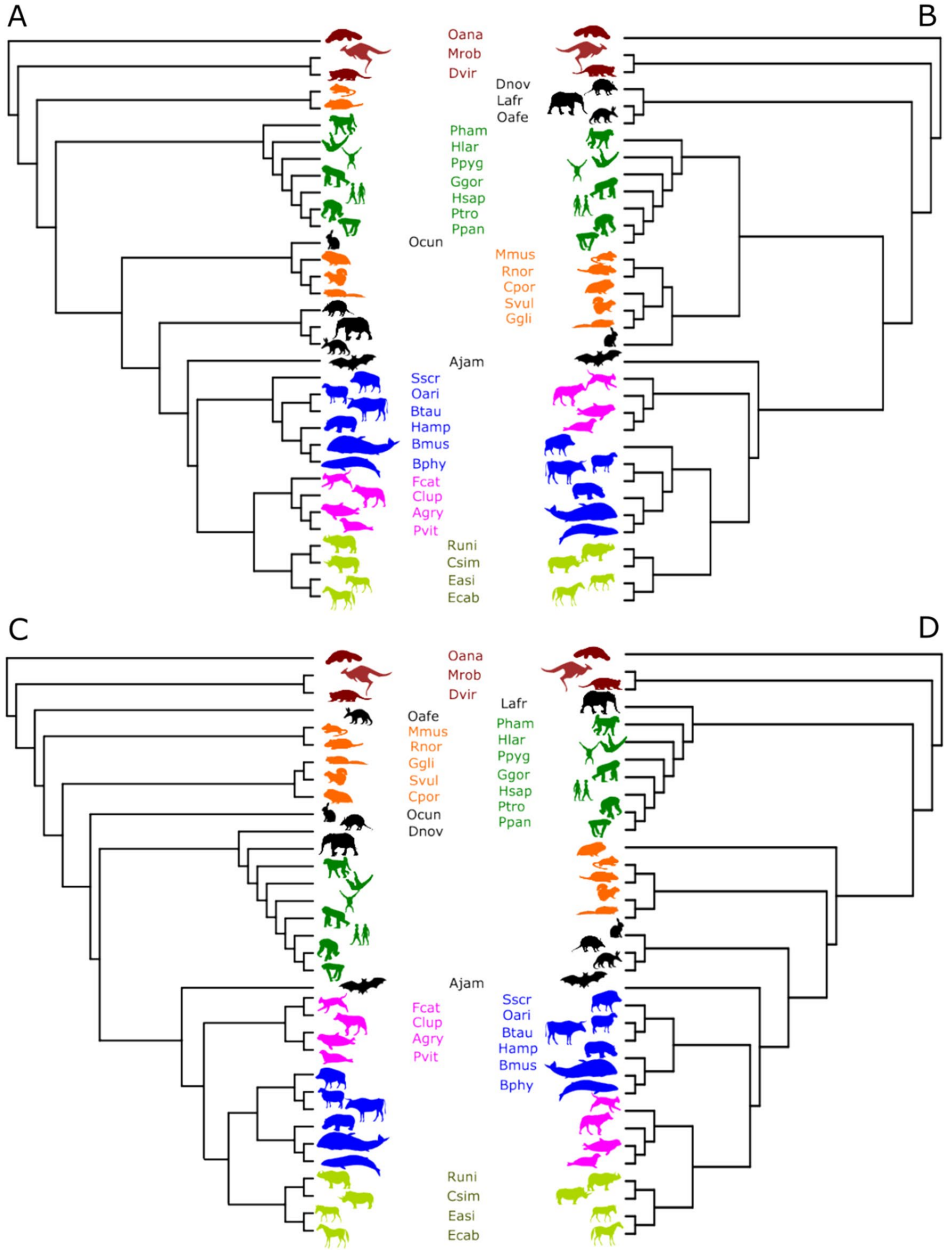
Comparison of phylogenetic tree topologies. The same mitogenomes of 34 mammalian species spanning 13 orders were employed with different tree-building methods. (**A**) Reproduces the topology of the tree obtained by Reyes et al. 2000 using a maximum likelihood approach based on multiple sequence alignments of nucleotides. (For protein genes, only first and second positions, P12, of the codons were considered. In addition, the ND6 gene encoded by the L-strand was also excluded). (**B**) The topology of the tree provided by VerLife^45^ for the relevant species is drawn. (**C**) The topology of the tree obtained using Env-NJ with a radius of 46 and the Jensen-Shannon metric is shown. (**D**) The topology of the tree constructed using singular value decomposition (SVD) to analyzed 4-mer string frequencies derived from unaligned sequences is also shown.

We next challenged the Env-NJ method with a larger, more diverse, and more controversial dataset consisting in the mitogenomes of 34 mammalian species spanning 13 orders. The phylogenetic relationships between the organisms of this set were first analyzed by Reyes and coworkers using a maximum likelihood approach^44^. Figure 3A reproduces the topology of the tree obtained by these authors. Nevertheless, since alternative hypotheses for the phylogeny of this set of species have been proposed by different authors^29,36^, in order to adopt a reference tree we resorted to the community resource VerLife^45^ to draw the topology of the tree (Fig. 3B) that relates the species under study according to this source^45^. As shown in Fig. 3, the Env-NJ method yields a credible phylogeny where primates, carnivores, cetartiodactyls and perissodactyls are some of the well-established mammalian lineages that appear as uninterrupted groupings within the Env-NJ tree. In order to obtain a more quantitative comparison, we next built 14 trees using the same dataset and different alignment-free tree building approaches. Afterwards, the normalized Robinson-Foulds symmetric difference between these trees and the reference tree was computed (Table 2). As it can be observed in this table, the Env-NJ with the Jensen-Shannon metrics provided the best result, understood as the one that provided the lowest Robinson-Foulds distance to the reference tree.

**Table 2:** Normalized Robison-Foulds distances between trees obtained using different alignment-independent methods with respect to the refence tree shown in Fig. 3B.

Approach	Package	Method	Language	nRF	Reference
Seq. environment	EnvNJ	cos, r = 46	R	0.39	Herein
Seq. environment	EnvNJ	jsd, r = 46	R	0.26	Herein
Seq. environment	EnvNJ	cheb, r = 46	R	0.61	Herein
k-mer counts	alfpy	cos, k = 5	Python	0.29	^25,27^
k-mer counts	alfpy	jsd, k = 5	Python	0.29	^25,27^
k-mer counts	alfpy	cheb, k = 5	Python	0.90	^25,27^
k-mer counts	FFP	jsd, k = 5	C	0.29	^26^
k-mer + f.p	^a^CVTree	cos, k = 5	C++	0.29	^32^
k-mer + f.p	^b^EnvNJ	SVD	R	0.29	^29^
k-mer + f.p	^c^alfpy	W-metric	Python	0.87	^25,27,33^
Matching statistics	ALFRED-G	cos, k = 5	C++	0.29	^35^
Splits/subseq	SANS-serif	strict, c = 2	C++	0.70	^38^
Information theory	alfpy	NCD	Python	0.29	^25,27^
Micro-alignments	Prot-SpaM	^d^(6, 40, 5)	C++	0.35	^39^

The whole protein-coding mitogenomes of a group of 34 mammalian species were analyzed. For details regarding the input parameters used for each method, please consult the given reference. Briefly, cos, jsd and cheb stand for cosine, Jensen-Shannon and Chebyshev metrics, respectively. The abbreviation f.p. stands for ‘further proccessing’. Thus (a) filters considering background words frequencies; (b) uses singular value decomposition, SVD, to analyze the 4-mer frequency data; (c) the matrix used for the W-metric analysis was Blosum62. (d) The parameters for the use of Prot-SpaM were weight of w = 6, with d = 40 (don’t-care positions) and m = 5 patterns. The parameter filter of SANS-serif was set to ‘strict’. NCD is the acronym of normalized compression distance.

**Figure 4:**
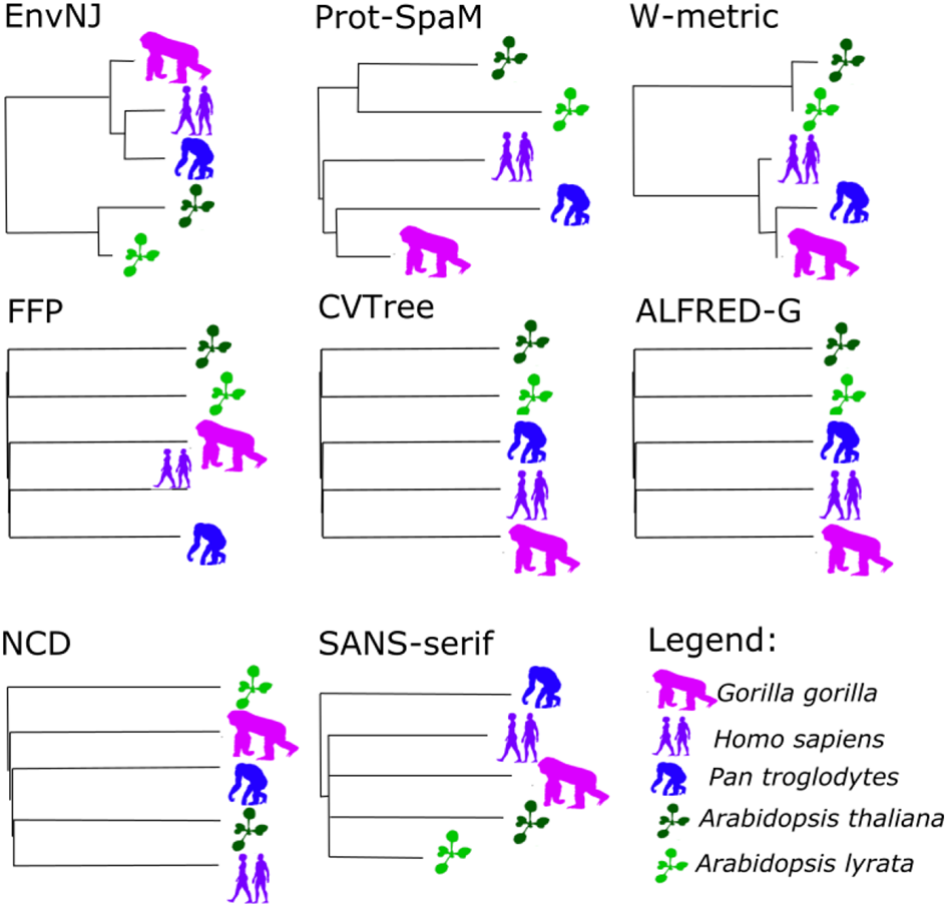
Alignment-free trees built using a dataset of non-orthologous protein sequences. For each species a random set consisting of 180 proteins was selected, with the only restriction that no protein belonging to this set could be homologous to any of the proteins belonging to the remaining species. These random sets of non-orthologous protein sequences were used to generate the corresponding trees.

At this point, we reason as follows. If the amino acid neighborhood preference in sequence environments is a species feature, then perhaps it may be possible to reconstruct phylogenetic relationships using non-orthologous proteins sets. To explore the potential of the Env-NJ method to provide such an achievement, we selected five species (three animals and two plants) whose phylogenetic relationships are well established. For each species a random set consisting of 180 proteins was selected, with the only restriction that no protein belonging to this set could be homologous to any of the proteins belonging to the remaining species. These random sets of non-orthologous protein sequences were used to generate the Env-NJ tree. To compare the performance of our method with that of previously proposed alignment-free approaches, the same dataset was subjected to 7 alternative methods, including Prot-SpaM^39^, W-metric^27,33^, FFP^26^, CVTree^32^, ALFRED-G^35^, normalized compression distances (NCD)^27^ and SANS-serfi^38^. As shown in Fig. 4, the new methodology yielded the correct tree topology even when non-orthologous proteins were employed, and under this specific conditions it seems to outperform other tree building methods.

Finally, all proteins encoded by the full genome of 11 plant species (Table 3) were used as input data to assess the performance of Env-NJ and other alignment-free alternative methods. The results are summarized in Table 4. As it can be observed in these tables, the Env-NJ approach was able to analyze 425,115 proteins accounting for 169,094,374 amino acids in less than 1 min, providing a reliable phylogeny.

**Table 3:** Plant dataset used to benchmark Env-NJ and other alignment-free approaches.

Species	Proteome ID	Number Proteins	Number Residues
*Arabidopsis lyrata*	UP000008694	32,113	11,683,288
*Arabidopsis thaliana*	UP000006548	39,328	16,643,018
*Brassica rapa*	UP000011750	40,809	15,960,268
*Capsella rubella*	UP000029121	28,039	11,560,300
*Citrus clementina*	UP000030687	31,273	12,651,335
*Citrus sinensis*	UP000027120	44,003	16,339,056
*Eucalyptus grandis*	UP000030711	44,150	16,844,312
*Eutrema salsugineum*	UP000030689	28,349	11,549,942
*Gossypium raimondii*	UP000032304	66,534	27,687,073
*Theobroma cacao*	UP000026915	40,611	17,402,995
*Vitis vinifera*	UP000009183	29,906	10,772,787

The FASTA files were downloaded from UniProtKB and used without further processing for establishing the phylogenetic relationship between the indicated species.

**Table 4:** Normalized (nRF) and generalized Robison-Foulds (GRF) distances between trees obtained using different alignment-independent methods with respect to the reference tree for the 11 plant species indicated in Table 3, which was obtained from AFproject^25^.

Package	Method	nRF	GRF	Time (secs)	Reference
EnvNJ	cos, r = 4	0.250	0.236	82	Herein
EnvNJ	jsd, r = 1	0.250	0.236	54	Herein
alfpy	cos, k = 5	0.625	0.475	126	^25,27^
alfpy	jsd, k = 5	0.125	0.127	125	^25,27^
alfpy	NCD	0.750	0.617	75	^25,27^
FFP	jsd, k = 5	0.875	0.583	732	^26^
CVTree	cos, k = 5	0.000	0.000	36	^32^
ALFRED-G	cos, k = 5	0.250	0.206	59	^35^
SANS-serif	strict, c = 2	1.000	1.000	8	^38^
Prot-SpaM	^a^(6, 40, 5)	0.000	0.000	42	^39^

For details regarding the input parameters used for each method, please consult the given reference. Briefly, cos and jsd stand for cosine and Jensen-Shannon metrics, respectively. (a) The parameters for the use of Prot-SpaM were weight of w = 6, with d = 40 (don’t-care positions) and m = 5 patterns. The parameter filter of SANS-serif was set to ‘strict’. NCD is the acronym of normalized compression distance. Runtimes were obtained with an Intel(R) Core(TM) i5-8600 CPU 3.10 GHz processor. Prot-SpaM was run on a Linus machine (Intel(R) Core™ i7-10700KF CPU 3.80 GHz processor).

## Discussion

Traditionally, the starting point to construct a molecular phylogeny has been identifying and gathering a set of evolutionary related (orthologous) sequences. However, before using these sequences to build a tree, it is important to ensure that each nucleotide or amino acid in each sequence is compared only with the corresponding homologous nucleotide or amino acid in the other sequences, what is referred to as positional homology. This preliminary task, that is one of the trickiest parts of the whole phylogenetic reconstruction process, is performed by aligning the sequences to one another. It should be noted that none of the frequently used alignment programs is capable of consistently producing perfect alignments, even when moderately divergent sequences are employed^46^. For that reason, it is always important to check the alignment quality before continuing with the phylogenetic reconstruction procedure. Obviously, this protocol is not scalable to phylogenomics. Since most genomes contain millions of sequence characters, these traditional methods based on positional homology comparisons, carried out over ambiguously resolved large-scale alignments, are unbusinesslike^14^. Thus, it seems that the problem of phylogenomic reconstruction based on site-evolution has no solution in the near future. To overcome this problem, different approaches have been explored.

One of these approaches to whole genomes phylogenetic analysis has focused on the ordering of the genes along the chromosomes, others have resorted to the gene content as its primary data. Since proteins (gene products) are modular and many of them are mosaics of diverse domains^47^, phylogenomic strategies based on domain-abundance or the presence/absence of protein folds may performe even better than those focused on genes^23,24^. The use of gene-order and gene/fold-content data in the context of phylogeny is the subject of important research efforts. However, there remain important challenges. Thus, mapping a full genome is a demanding task. Furthermore, the posterior analysis of the annotated genome is computationally expensive and time consuming because of the extreme mathematical complexity of gene orders. For instance, for a chromosome with *n* distinct single-copy genes, the number of possible states is ${2}^{n-1}\left(n-1\right)!$^48^. This computational burden means that all reconstruction methods face a considerable challenge, even on small datasets consisting of only a few genomes. Furthermore, in these approaches the information contained into a genome is largely simplified, in the sense that point mutations are completely ignored, that is, these methods somehow make use of lossy data compression, so that relevant information contained in a genome is not used to infer its evolutionary history.

In this report, we describe a phylogenetic approach based on sequence environments, that may be valuable for the future development of new methods for generating phylogenies from whole genomes without resorting to lossy data compression. The protein-coding genomes of the set of organisms being analyzed are converted into a matrix, where each column vector represents a species. More concretely, these vectors represent the species-specific amino acid neighborhood preferences (Fig. 1). During our previous investigations, we had observed that different species exhibited a differential preference for amino acids in the vicinity of their methionine residues, even though the relative frequencies of the proteinogenetic amino acids were very similar in the analyzed species. This observation prompted us to explore the potential of sequence environments to accurately reconstruct phylogenies using genome/proteome datasets of unaligned sequence information.

As a first approach, we decided to carry out a pilot study using an optimal set of genomes, in the sense that the expected tree topology is widely accepted. To this end, we chose the protein-coding mitogenomes of 11 species of bovids. This dataset is small and simple, coding for only 143 proteins whose sequences are curated by the NCBI and are expected to be very accurate. Furthermore, the orthologous relationships among the proteins belonging to this set are obvious and undisputed. Moreover, mitochondrial sequences are often used to generate metazoan phylogenies, hence the tree generated by Env-NJ can be easily compared to those generated by other methods, either based on sequence alignments or alignment-independent. Given that the group of organisms was formed by species closely related, and the optimal conditions discussed above, not surprisingly, most methods consistently produced the same tree topology (Fig. 2 and Table 1).

Encouraged by this success, we next tested the Env-NJ method with a larger, more diverse and more controversial dataset consisting in the mitogenomes of 34 mammalian species spanning 13 orders. This dataset, first analyzed by Reyes and coworkers using alignment-based methods^44^, has been used later by different authors employing different tree building methods. Thus, the same genome set has been analyzed by Stuart and coworkers using the SVD-4-Gram method^29^ and also by Li and colleagues, using a method that also works on unaligned sequences, but in this case exploiting the Kolmogorov complexity concept to estimate distances between genomes^36^. In the R package *EnvNJ* accompanying the current paper (throughout the text we use the term Env-NJ to indicate the method, while *EnvNJ* refers to the software) we have implemented, in addition to the Env-NJ method, those utilities required to reproduce the trees reported by Reyes et al. (Fig. 3A) and Stuart and coworkers (Fig. 3D), which are shown herein for comparative purposes. The method based on the Kolmogorov complexity was not included in the comparison because, although it faithfully reproduced the tree obtained by Cao and coworkers for a smaller and less conflictive set of mammalian species^3^, it offered a rather poor phylogeny, showing polytomy, for the taxa we are addressing herein (see Fig. 2 from Li et al. 2001).

Figure 3 and Table 2 summarize the topologies comparison of the trees obtained with the different methods being compared. Overall, the Env-NJ tree seems to be a reasonably good approximation to the reference tree, at least as good as any of the trees obtained by alternative methods. This was also true when mitogenomes from other groups of vertebrates were analyzed (Fig. S1). To this respect, a group of 25 species of fish, which is widely used to benchmark alignment-independent phylogenetic methods^25^, was analyzed using different approaches. Again, the Env-NJ yielded excellent results, both in term of computation time as well as regarding tree-topology reliability.

**Figure 5:**
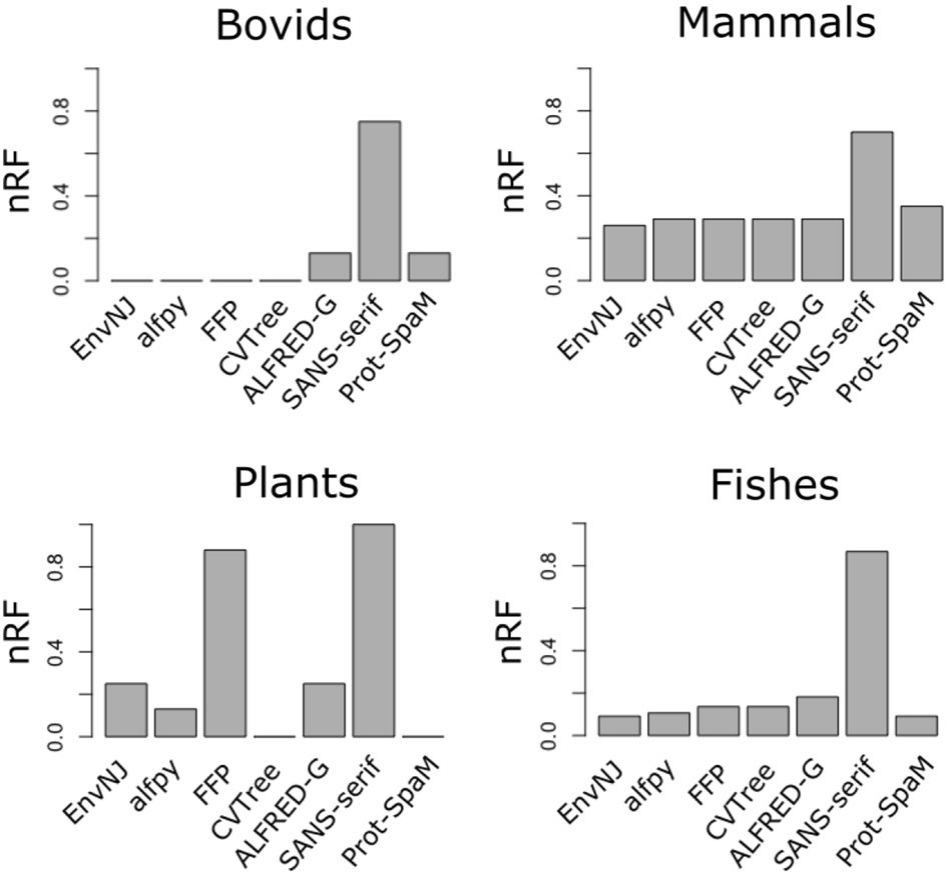
Summary of the performance of different programs with different data sets. Four sets of sequences were analyzed with the programs EnvNJ, alfpy, FFP, CVTree, ALFRED-G, SANS-serif and Prot-SpaM, using different parameters combinations. The perform of each method, in terms of nRF distances to the reference tree is given in Table 1 for the bovid group, Table 2 for the group of mammals, Table 4 for the plant species and Figure S1 for the fish species. This figure summarizes and shows the results that each program yielded with the optimal selection of parameters.

In the current work, the accuracy of the different alignment-independent methods has been evaluated by a comparison of topology between the reconstructed tree using a given method and the corresponding reference tree. For this purpose, we computed the nRF distance between the trees, which is a straightforward to interpret metric (Fig. 5). Despite of being a metric widely used in the literature to quantify similarity between pairs of phylogenetic trees^25,26,35,39,40,49,50^, the nRF is known to present certain shortcomings such as rapid saturation and imprecise values^51,52^. Therefore, to rule out that these drawbacks could be biasing the results presented herein, we also computed a generalized RF metric (GRF) designed to avoid the limitations of the nRF^53^. Using two different datasets (Fig. S1 for the fish group, and Table 4 for the plant group) we found a good positive correlation between nRF and GRF (R-squared = 0.97, p-value = 2.4 10^–9^), and the conclusions obtained regarding the benchmark analyses are equally well supported regardless the metric employed.

We have extensively assessed the performance of the Env-NJ method on mitogenomes. In this context, the new method seems to be a valid alternative for phylogenomics since it has three valuable properties: (i) accuracy, (ii) speed and (iii) independence of positional homology. Indeed, Env-NJ does not rest on positional homology, and it does not require to identify orthologous proteins to proceed with the computation. However, one thing is that the method does not require identification of orthologous proteins, and quite another is that the method does not require the presence of orthologous proteins in the dataset. The latter is guaranteed when working with mitogenomes, where the presence of one-to-one orthologous proteins is guaranteed. On the other hand, when all proteins encoded by the full genome of the analyzed species are used as input, the success of the Env-NJ approach (Table 4) could be due to the presence of a high proportion of orthologous proteins in the input dataset. Therefore, we next wondered whether the Env-NJ method would be able to reconstruct a phylogeny analyzing non-orthologous proteins? That is, when each species contributes a set of proteins completely unrelated to the protein sets contributed by the other species under analysis.

To address this issue, we chose a small set of species formed by three animals (human, chimp and gorilla) and two plants (*Arabidopsis thaliana* and *A. lyrate*). For each species we randomly sampled 180 protein sequences from its proteome. The selection process was random with the only restriction that there were no pairs of orthologous proteins among the 900 sequences that made up the dataset (both the script to sample the sequences and the sequences themselves can be obtained at https://bitbucket.org/jcaledo/envnj/src/master/AncillaryCode/Oma_PlantAnimal.R, and https://bitbucket.org/jcaledo/envnj/src/master/Datasets/oseq.Rda, respectively). When this dataset was subjected to Env-NJ, the recovered tree was the expected one (Fig. 4), where human was closer to chimp than gorilla, and the two plants appeared as sister operational taxonomic units (OTUs). More interestingly, under these challenging conditions, the strategy based on sequence environments was the only one that provided an acceptable result. Having a tree building method that does not require orthologs identification is a good thing, and Env-NJ is indeed such a method. Having a method that does not even require the presence of orthologs in the dataset, is even better and Env-NJ may fulfil this feature. A drawback of Env-NJ, as well as most alignment-free approaches, is that they are distance methods. That is, there is no evolutionary model behind them, which precludes the use of maximum likelihood techniques to explore tree spaces. Undoubtedly, further research effort will be required before we can witness a significant breakthrough in the field of phylogenomics.

## Conclusion

In the current report we describe a new tree-building method and its implementation into an R package (*EnvNJ*). This new method presents many advantages: (i) it does not resort to lossy data compression; (ii) it is computationally very fast, making it suitable for addressing whole genomes; (iii) because the method makes use of whole genomes/proteomes, there is no gene tree versus species tree problem; (iv) there is no need for multiple sequence alignment, which contributes to the speed of the method and avoids the impact of misalignments on the tree topology; (v) it does not require orthology identification, which further contributes to shortening computation times. Finally, the possibility that Env-NJ may perform well even with non-orthologous protein datasets, is a line of research that deserves further work in the future.

## Material and methods

### The species vector space

It has been shown that every amino acid has a characteristic sequence environment in proteins^41,54^. In previous works, we have analyzed the sequence environment (10 residues on each side) around methionine residues in human proteins^42,43,55^. Thus, and just based on methionine residues, the human proteome can be characterized by a matrix, **M**, whose elements (m_ij_) provide the absolute frequency of the amino acid *i* at the position *j* in the environment of methionine residues (Fig. 1A). Similarly, for each proteinogenic amino acid, $X\in \left\{A,R,N,D,C,Q,E,G,H,I,L,K,M,F,P,S,T,W,Y,V\right\}$, a matrix $\left({x}_{ij}\right)\in {\mathcal{M}}_{20}({\mathbb{N}})$ can be computed. In this way, the protein-coding genome of a given species of interest can be characterized by a set of 20 square matrices of order 20 or, equivalently, by a vector, $u\in {U}^{8000}$, of dimension 8000 (Fig. 1B). However, when coding species as vectors, the dimension of these vectors will depend on the radius chosen. Thus, in general, $u\in {U}^{n}$, where $n=20 \left(2 radius\right) 20=800 radius$. In this way, each vector is used to represent an organism (its protein-coding genome). The components (coordinates) of the vector reflect the preference of the different amino acids at the different positions of the sequence environments.

### A suitable metric for the species vector space

Once species are encoded as high-dimensional vectors, we can make use of the extensive mathematical tools of numerical linear algebra. Since we are interested in assessing distances between species, we must endow this vector space with a suitable metric for our purpose. To this end, we must look for functions, *d*, able to provide a distance between vectors.

$$d:{U}^{n}x{U}^{n}\to {\mathbb{R}}$$

In general, any function, *d*, to be considered a distance must satisfy the following 4 properties. (i) Positive definiteness: $d\left({u}_{i},{u}_{j}\right)\ge 0 \forall {u}_{i},{u}_{j}\in {U}^{n}$; (ii) coincidence axiom: $d\left({u}_{i},{u}_{j}\right)=0\Leftrightarrow {u}_{i}={u}_{j}$; (iii) symmetry: $d\left({u}_{i},{u}_{j}\right)=d\left({u}_{j},{u}_{i}\right) \forall {u}_{i},{u}_{j}\in {U}^{n}$; and (iv) triangle inequality: $d\left({u}_{i},{u}_{j}\right)\le d\left({u}_{i},{u}_{k}\right)+d\left({u}_{k},{u}_{j}\right) \forall {u}_{i},{u}_{j},{u}_{k}\in {U}^{n}$. A wide variety of metrics can be used to measure relatedness between vectors. The function vect2tree() from the *EnvNJ* package accompanying this paper, implements 29 different metrics previously described^56,57^. However, as illustrated in Fig. 1C,D, not all of them will be equally suitable for our purpose of establishing evolutionary relationships between species. Furthermore, the link between a given metric and its performance is not always obvious^58^. Nevertheless, for the sequence datasets we have used in the current study, we have noticed that the so-called Jensen-Shannon and cosine-based dissimilarities perform better than other metrics. Although many of the offered methods compute proper distances, that is not always the case. For instance, the ‘cosine’ method we described next, does not satisfy the coincidence axiom, so it cannot be considered a true distance. This fact, far from being a drawback, can be an advantage (as it will be argued below) for our phylogenetic purposes. In the context of latent semantic analysis, a common measure of similarity between two vectors is the cosine of the angle between them^58,59^. Since protein sequence data can be regarded as a complex written language, Stuart and coworkers have proposed the use of the cosine between two vectors as a suitable measure of vector similarity when the vectors being considered contain information related to protein sequences^28,29^. For instance, if we have the protein-coding genome of the species *i* and *j* (Fig. 1C), their similarity can be assessed by the expression:

$$cos{\theta }_{ij}=\frac{{u}_{i}^{T}{u}_{j}}{\Vert {u}_{i}\Vert \Vert {u}_{j}\Vert }$$

where ${u}_{i}^{T}{u}_{j}$ is the dot product of the vectors ${u}_{i}$ and ${u}_{j}$, and $\Vert \Vert$ is the Euclidean vector norm. It should be noted that the function $f\left({u}_{i},{u}_{j}\right)=cos{\theta }_{ij}$ is not a distance properly speaking. For instance, suppose that two species have identical genomes, in this case ${u}_{i}={u}_{j}$ and we would expect a null distance between them. However, $f\left({u}_{i},{u}_{j}\right)=cos{\theta }_{ij}=cos0=1$. Nevertheless, pairwise cosine values can be converted into pairwise evolutionary distances using the following formula:

$$d\left({u}_{i},{u}_{j}\right)=-ln\frac{1+cos{\theta }_{ij}}{2}$$

This formula converts a similarity measure into a distance measure^28^. It is important to note that this evolutionary distance is not a proper distance metric as it violates the coincidence axiom. For instance, $d\left({u}_{i},{2u}_{j}\right)=0$ but ${u}_{i}\ne 2{u}_{i}$. However, this violation is very convenient for our goals. A concrete example will be useful to understand this assertion. Suppose that we have a population that splits into two species. Suppose, further, that one of this species undergoes a genome duplication event, but otherwise their proteomes are identical. In such a scenario, the computed species vectors would be ${u}_{j}=2{u}_{i}$. Although both vectors have different lengths, since their directions are identical, we obtain $d\left({u}_{i},{u}_{j}\right)=0$, which conveniently reflects the fact that their proteomes are equal and therefore the neighborhood preferences of their sequence environments are the same in both species (Fig. 1D).

### Environment-based trees

After encoding the genome of each species into a vector, as described above, these vectors are used to obtain a matrix of pairwise cosine values that are subsequently converted into a matrix of pairwise evolutionary distances using the formula given in the previous section. Alternatively, other metrics can be used to obtain a distance matrix. In the *EnvNJ* package accompanying this paper, we have implemented 29 different metrics among which the user can choose. However, in our experience the cosine-based dissimilarity and the Jensen-Shannon distance are among the best performing metrics for phylogenetic analyses using sequence environments. In any case, the obtained distance matrix can be used to produce a phylogenetic tree employing the neighbor joining algorithm^60^.

### Implementation

The Env-NJ tree building method has been implemented in an R package, *EnvNJ*. The package, which works on all major operating systems (Windows, MacOS and Linux) can be installed either from CRAN, *install.packages(“EnvNJ”)*, or from its bitbucket repository, typing consecutively the following three commands in an R terminal: *install.packages(“devtools”), library(devtools), install_bitbucket(“jcaledo/envnj”, subdir* = *“REnvNJ”)*. Since the protein sequence datasets analyzed in the current work (see below) have also been included into the package, once it has been installed, the trees shown in Fig. 2 and 3C can be easily obtained with the commands *envnj(bovids, r* = *2)* and *envnj(reyes, r* = *46)*, respectively. Further help can be obtained from the package documentation by introducing into the R terminal: *?envnj*. A vignette about the use of the *EnvNJ* package can be found as Supplementary Material.

In addition to the Env-NJ method described in this paper, the package *EnvNJ* also implements the method based on the SVD-n-Gram approach, previously described by Stuart and coworkers^28^. The aim was to facilitate its use for comparative purposes (check the documentation, *?svdgram*).

### Mitogenome Datasets

The mtDNA-encoded protein sequences were obtained from the NCBI genome database (https://www.ncbi.nlm.nih.gov/genome/organelle). Two sets of mitogenomes have been analyzed in the current work. The first set is formed by 11 species of bovids including *Bison bison, Bison bonasus, Bos grunniens, Bos indicus, Bos javanicus, Bos primigenius, Bos taurus, Bubalus bubalis, Bubalus depressicornis, Pseudoryx nghetinhensis, Syncerus caffer*. An R dataframe containing these sequences can be loaded and examined by typing *data(bovids)* after having installed the R package *EnvNJ*.

A second set of mitogenomes analyzed in this study is the one formed by 34 mammalian species spanning 13 orders, first used by Reyes and coworkers^44^, which includes the following species: *Artibeus jamaicensis* (Ajam), *Balaenoptera musculus* (Bmus), *Balaenoptera physalus* (Bphy), *Bos taurus* (Btau), *Canis lupus* (Clup), *Cavia porcellus* (Cpor), *Ceratotherium simum* (Csim), *Dasypus novemcinctus* (Dnov), *Didelphis virginiana* (Dvir), *Equus asinus* (Easi), *Equus caballus* (Ecab), *Felis catus* (Fcat), *Glis glis* (Ggli), *Gorilla gorilla* (Ggor), *Halichoerus grypus* (Hgry), *Hippopotamus amphibius* (Hamp), *Homo sapiens* (Hsap), *Hylobates lar* (Hlar), *Loxodonta africana* (Lafr), *Macropus robustus* (Mrob), *Mus musculus* (Mmus), *Ornithorhynchus anatinus* (Oana), *Orycteropus afer* (Oafe), *Oryctolagus cuniculus* (Ocun), *Ovis aries* (Oari), *Pan paniscus* (Ppan), *Pan troglodytes* (Ptro), *Papio hamadryas* (Pham), *Phoca vitulina* (Pvit), *Pongo pygmaeus* (Ppyg), *Rattus norvegicus* (Rnor), *Rhinoceros unicornis* (Runi), *Sciurus vulgaris* (Svul), *Sus scrofa* (Sscr). Again, this dataset can be obtained in a suitable format (as dataframe) by typing in the R terminal: *data(reyes).*

### Env-NJ trees using non-orthologous protein datasets

We chose three closely related primate species (human, chimp and gorilla) and two Arabidopsis species (*A. thaliana* and *A. lyrate*). Since we wanted to make sure that no orthology could be established between any pair of proteins from the dataset subjected to analysis, we proceeded as described next. First, we started by identifying a set of 907 one-to-one orthologous proteins present in the five species. To achieve that, we took advantage of the REST API for the OMA orthology database^61,62^. Both, the dataset (oseq.Rda) and the script (Oma_PlantAnimal.R) used to obtain it, can be downloaded from https://bitbucket.org/jcaledo/envnj/src/master/Datasets and https://bitbucket.org/jcaledo/envnj/src/master/AncillaryCode, respectively. In this way, the oseq.Rda object is a dataframe with five columns (one per species) and 907 rows (one per orthologous protein), and each entry contains the corresponding protein sequence. To form a dataset of non-orthologous proteins we proceeded as follows. For the first column (species) we randomly chose 180 rows (proteins). Afterward, the randomly selected rows were discarded from the dataframe before proceeding with the next column (species). Among the remaining rows, again we randomly selected 180, and the corresponding proteins from the second species were selected before removing the randomly selected rows from the dataframe. This operation was repeated until reaching the last species, at which point we had a collection of 900 non-orthologous proteins (180 per species). This randomly selected dataset formed by 900 non-orthologous proteins was then subjected to Env-NJ.

### Plant proteomes

AFproject (http://afproject.org) is a publicly available web-based service for objective performance comparison of alignment-free sequence comparison tools on different datasets^25^. They provide a benchmark dataset formed by the full genome sequences for 14 plant species and the corresponding reference species tree. Since the Env-NJ approach uses protein sequences and to avoid pre-processing (identification of open reading frames and translation) we resorted to the UniProt Proteomes (https://www.uniprot.org/proteomes) to search for protein sequences belonging to this group of plant species. In this way, we managed to assemble a dataset formed by 425,115 proteins from 11 species accounting for around 170 million amino acids (Table 3). The three species for which we could not find enough data were pruned from the reference tree.

### Robinson–Foulds distance

As a measure of the accuracy attributable to each phylogeny, the normalized Robinson-Foulds (nRF) distances between the reconstructed trees and the reference trees were computed. The Robinson-Fould algorithm to compute distances between trees topologies^63^, as implemented in the R package phangorn^64^, was used for this purpose. Briefly, Let T_1_ and T_2_ be two sets formed by all the splits at internal edges for tree 1 and tree 2, respectively (the two trees whose topologies we want to compare), then the cardinal of the symmetric difference of these two sets provides the Robinson-Foulds distance.

$$RF= \left|{T}_{1}\Delta {T}_{2}\right|$$

In other words, the Robison-Foulds is the number of splits appearing in one tree but not the other. The normalized Robinson-Foulds distance, nRF, is obtained by dividing RF by the maximal possible distance, that is

$$nRF= \frac{\left|{T}_{1}\Delta {T}_{2}\right|}{\left|{T}_{1}\right|+ \left|{T}_{2}\right|}$$

Normalization forces this metric to take values between 0 and 1, which makes its interpretation straightforward: 0 indicating identical tree topologies and 1 pointing to the most dissimilar topologies.

## Supplementary Information

Supplementary Information 1.

Supplementary Information 2.

## Data Availability

The Env-NJ method is implemented in the R package *EnvNJ.* Release versions are available via CRAN and work on all major operating systems. The development version is maintained at https://bitbucket.org/jcaledo/envnj/src/master. The mtDNA-encoded protein sequences in the 11 species of bovids can be obtained from the *EnvNJ* package just typing, after loading the package, *data(bovids)*. Similarly, the 442 protein sequences that make up the dataset referred to as Reyes, can be obtained typing *data(reyes).* Alternatively, all the data employed in the current work, together with their corresponding descriptions can be obtained from the Bitbucket repository at https://bitbucket.org/jcaledo/envnj/src/master/Datasets.
