# Supplementary material for: Phylogenies from unaligned proteomes using sequence environments of amino acid residues
Source: Sci Rep. 2022 May 6;12:7497. doi: 10.1038/s41598-022-11370-x (PMC9076898; doi:10.1038/s41598-022-11370-x)

Supplementary Figure S1

**Phylogenies from unaligned proteomes using sequence environments of amino acid residues**

Juan Carlos Aledo

Department of Molecular Biology and Biochemistry, University of Málaga, 29071-Málaga, Spain.

**Figure S1**. Comparison of phylogenetic tree topologies. The amino acid sequences of the 13 proteins encoded into the mtDNA of a group of 25 fish species were downloaded from <https://www.ncbi.nlm.nih.gov/genome/organelle/> and employed with different tree-building methods. The analyzed species were:

*Abudefduf vaigiensis* (NC_009064), *Alepocephalus agassizii* (NC_013564),

*Amphiprion ocellaris* (NC_009065), *Astronotus ocellatus* (NC_009058),

*Bajacalifornia megalops* (NC_013577), *Cymatogaster aggregata* (NC_009059),

*Ditrema temminckii* (NC_009060), *Etroplus suratensis* (NC_029832),

*Halichoeres melanurus* (NC_009066), *Hypselecara temporalis* (NC_011168),

*Neolamprologus brichardi* (NC_009062), *Oreochromis aureus* (NC_013750),

*Oreochromis niloticus* (NC_013663), *Oreochromis sp-KM2006* (NC_009057),

*Parajulis poecilepterus* (NC_009459), *Paratilapia polleni* (NC_011170),

*Paretroplus maculatus* (NC_011177), *Petrochromis trewavasae* (NC_018814),

*Pseudolabrus eoethinus* (NC_012055), *Pseudolabrus sieboldi* (NC_009067),

*Pteragogus flagellifer* (NC_010205), *Ptychochromoides katria* (NC_011169),

*Tropheus duboisi* (NC_009063), *Tropheus moori* (NC_018815),

*Tylochromis polylepis* (NC_011171).

On the other hand, the AFproject resource at <http://afproject.org/app/benchmark/genome/std/assembled/fish_mito/results> provided a reference tree for this group of fishes, with an exception: *Etroplus maculatus* (for whose species it was not possible to find its mitogenome in NCBI) was replaced by *Etroplus suratensis.* The reference tree used, in Newick format, was: ((Alepocephalus_agassizii,Bajacalifornia_megalops),

(((Parajulis_poecilepterus,Halichoeres_melanurus),

(Pteragogus_flagellifer,(Pseudolabrus_eoethinus,Pseudolabrus_sieboldi))),

(((Ditrema_temminckii,Cymatogaster_aggregata),

(Abudefduf_vaigiensis,Amphiprion_ocellaris)),

((Etroplus_suratensis,Paretroplus_maculatus),

((Paratilapia_polleni,Ptychochromoides_katria),

((Hypselecara_temporalis,Astronotus_ocellatus),

(Tylochromis_polylepis,((Oreochromis_sp-KM2006,

(Oreochromis_aureus,Oreochromis_niloticus)),

(Neolamprologus_brichardi,(Petrochromis_trewavasae,

(Tropheus_moorii,Tropheus_duboisi)))))))))));+

In the figure below, the normalized and generalized Robinson-Faulds distances (nRF and GRF, respectively) are given. The runtimes using an iMac with an Intel(R) Core(TM) i5-8600 CPU 3.10GHz processor, are also shown.


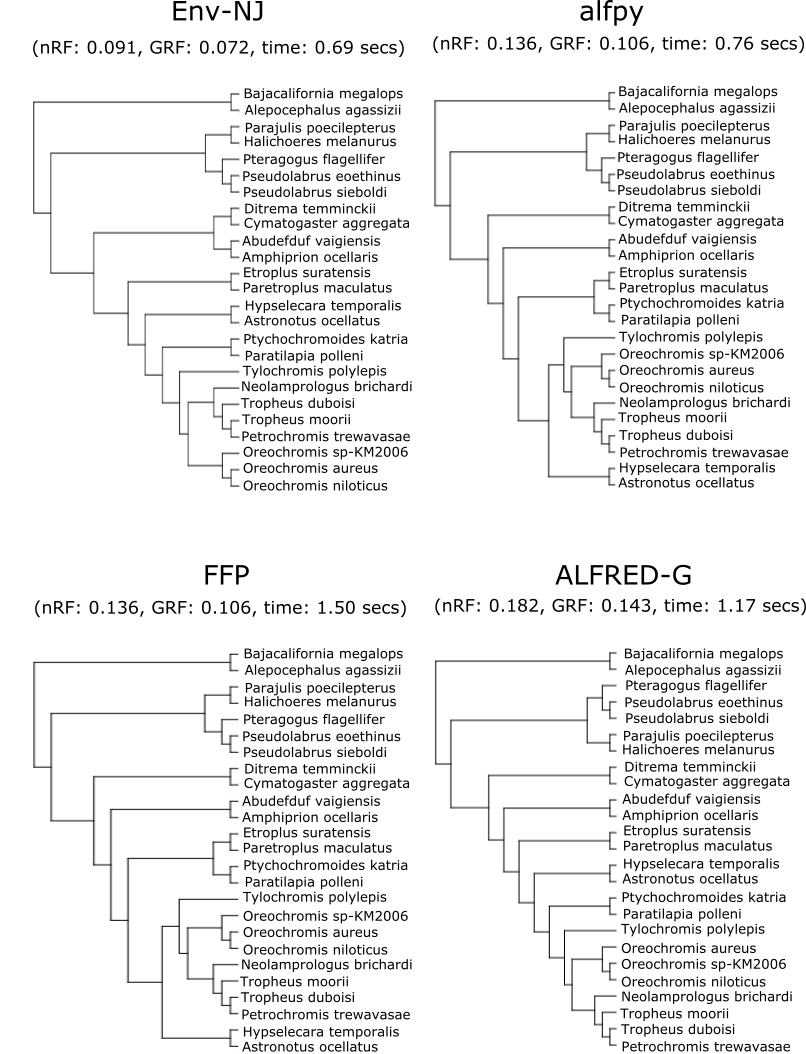

Supplement: Supplementary file 1 — Supplementary Information 1. [file 41598_2022_11370_MOESM1_ESM.docx]
