# Supplementary material for: Phylogenies from unaligned proteomes using sequence environments of amino acid residues
Source: Sci Rep. 2022 May 6;12:7497. doi: 10.1038/s41598-022-11370-x (PMC9076898; doi:10.1038/s41598-022-11370-x)

How to use EnvNJ

Juan Carlos Aledo

How to install EnvNJ

Before you attempt to install **EnvNJ** you should have a relatively recent version of R (4.0.0 or higher) installed and working on your system. Detailed instructions for obtaining and installing R on various platforms can be found on the R home page. We also encourage you to install RStudio, a useful integrated development environment for R.

On all plataforms (Mac, Windows and Linux) open R and use the function `instal.packages()` at the R command prompt to install the package from your local CRNA site:

```
install.packages("EnvNJ")
```

Alternatively, if your prefer to use RStudio, select Tools -> Install Packages .... Enter **EnvNJ** in the Packages text box and then click Install.

Another alternative is to install **EnvNJ** using its Bitbucket repository and the R function `install_bitbucket()` from the devtools package.

```
install.packages("devtools")
library(devtools)
install_bitbucket("jcaledo/envnj", subdir = "REnvNJ")
```

Optional Dependencies

Although **EnvNJ** is an R package aimed to generate alignment-independcent phylogenetic trees, occasionally, the user may wish to compare such trees with those obtained using traditional methods based on multiple sequence alignments. Thus, **EnvNJ** includes functions to obtain these trees. However, for that purpose, you should have MUSCLE installed on your system and in the search path for executables. MUSCLE is a fast multiple sequence alignment program available from the MUSCLE home page.

For quick install on MacOS we recommend using homebrew. In a MacOS terminal prompt, paste:

```
/usr/bin/ruby -e "$(curl -fsSL https://raw.githubusercontent.com/Homebrew/install/master/install)"
```

Then, type:

```
brew install brewsci/science/muscle
```

On a Linux/UNIX system you should use your appropiate package manager (e.g. apt-get for Debian/Ubuntu and nf for Red Hat/Fedora systems). If you experience problems with any of these steps, please read on for alternative installation methods.

Bovids Phylogeny

The package **EnvNJ** includes data (mtDNA-encoded protein sequences) from 11 species of bovids. This data can be loaded as follows:

```
library(EnvNJ)
data(bovids)
```

If you eximine the loaded data, you will note that is a dataframe with 13 rows (one per mtDNA-encoded protein) and 11 columns (one per species). That is the data structure required to use the function `envnj()` that will built a tree in an alignment-independent and fast way:

```
Env_result <- envnj(bovids)
```

The obtained object, that we have decided to name `Env_result`, is actually a list where the first element is a distance matrix and the second element (`Env_result[[2]]`) is the unrooted tree that has been built using those distances. To root the tree we are going to use the function `midpoint()` from the package **phangorn**. The next chunk will install that package if needed and will root the tree.

```
if (!requireNamespace("phangorn", quietly = TRUE)){
  install.packages()
}

Env_tree <- phangorn::midpoint(Env_result[[2]])
```

Now we can plot the tree using the suitable function from the package **ape**:

```
ape::plot.phylo(Env_tree, use.edge.length = FALSE)
```

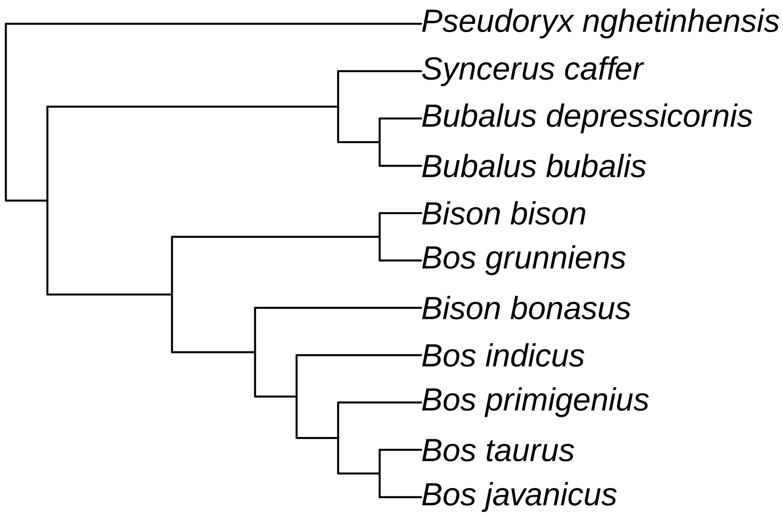

For comparative purposes, next, we are going to use the same dataset but different methods to reconstruct the bovids phylogeny. Let's start using another method that also works on unaligned sequences. This method is based on the 4-gram frequencies analysis. See Bionformatics 18, 100-108 for a detailed description of the method.

First, we are going to compute the tetrapeptide frequencies. Please, note that this task can take a few minutes:

```
pept_frequencies <- ngraMatrix(bovids, k = 4)[[1]]
```

Afterward, we can build the tree using the computed tetrapeptides frequencies:

```
svd_tree <- svdgram(pept_frequencies[, -1], rank = 40, species = names(bovids))
svd_tree_rooted <- ape::root(svd_tree[[1]], outgroup = "Pseudoryx_nghetinhensis", resolve.root = TRUE)
ape::plot.phylo(svd_tree_rooted, use.edge.length = FALSE)
```

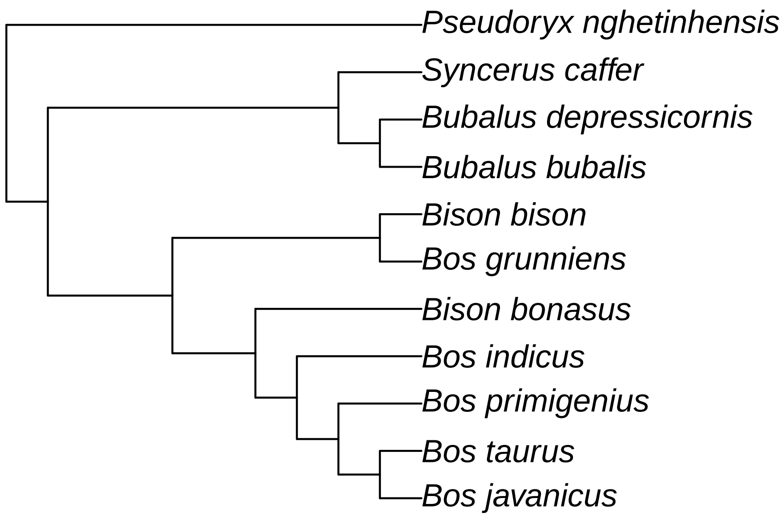

Finally, we are going to repeat the process of reconstructing the phylogenetic relationships betwee these species of bovids, but now using a traditional method (neighbor joining) based on sequence alignment:

```
nj_tree <- msa.tree(bovids)
nj_tree_rooted <- phangorn::midpoint(nj_tree[[3]])
ape::plot.phylo(nj_tree_rooted, use.edge.length = FALSE)
```

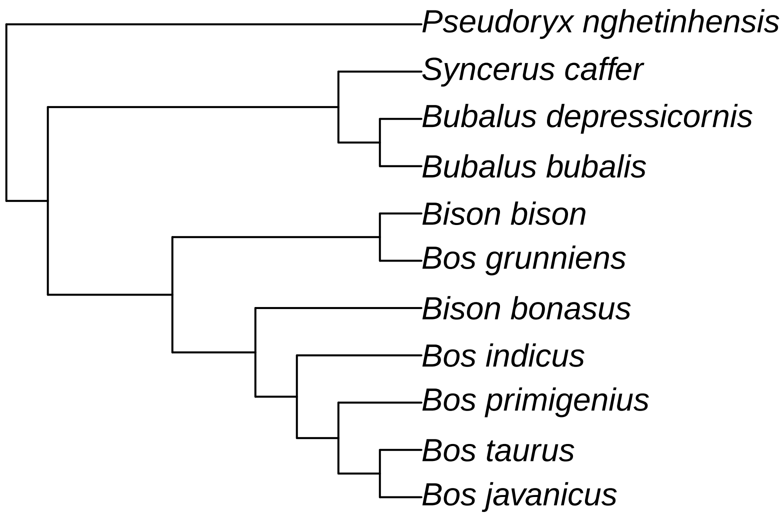

Supplement: Supplementary file 2 — Supplementary Information 2. [file 41598_2022_11370_MOESM2_ESM.pdf]
